# The combination of RNA-seq transcriptomics and data-independent acquisition proteomics reveals the mechanisms underlying enhanced salt tolerance by the *ZmPDI* gene in *Zoysia matrella* [L.] Merr.

**DOI:** 10.3389/fpls.2022.970651

**Published:** 2022-08-08

**Authors:** Qiang Ming, Kai Wang, Jingjing Wang, Jianxiu Liu, Xiaohui Li, Peipei Wei, Hailin Guo, Jingbo Chen, Junqin Zong

**Affiliations:** ^1^The National Forestry and Grassland Administration Engineering Research Center for Germplasm Innovation and Utilization of Warm-season Turfgrasses, Institute of Botany, Jiangsu Province and Chinese Academy of Sciences, Nanjing, China; ^2^Jiangsu Coastal Area Institute of Agricultural Sciences, Yancheng, China

**Keywords:** *Zoysia matrella*, salt tolerance, PDI, RNA-seq, proteome

## Abstract

*Zoysia matrella* [L.] Merr. is one of the three most economically important *Zoysia* species due to its strong salt tolerance and wide application. However, the molecular mechanisms regulating salt tolerance in *Z. matrella* remain unknown. The protein disulfide isomerase *ZmPDI* of *Z. matrella* was obtained by salt stress screening with yeast cells, and its expression was significantly upregulated after salt stress. Based on the obtained *ZmPDI* overexpression transgenic *Z. matrella* plants, we carried out salt tolerance identification and found that *ZmPDI* can significantly enhance the salt tolerance of *Z. matrella*. Root samples of *OX-ZmPDI* transgenic and wild-type plants were collected at 0 and 24 h after salt treatments for RNA-seq and data-independent acquisition (DIA) proteome sequencing. Combined analysis of the transcriptome and proteome revealed that *ZmPDI* may enhance the salt tolerance of *Z. matrella* by regulating *TUBB2*, *PXG4*, *PLD*α*2*, *PFK4*, and *4CL1*. This research presents the molecular regulatory mechanism of the *ZmPDI* gene in *Z. matrella* for resistance to salt stress and facilitates the use of molecular breeding to improve the salt tolerance of grasses.

## Introduction

Saline-alkali land is widely distributed throughout the world and seriously affects the local ecological environment and economic development. How to efficiently use and improve saline-alkali land resources has become a global research hotspot. Turfgrass plays an important role in ecological and green space construction and is a pioneer plant for ameliorating soil salinization. *Zoysia* grass is an excellent warm-season turfgrass that is famous for its strong tolerance (including salt, cold, and drought tolerance) and wide application (including saline-alkali soil, athletic fields, home lawns, and parks) (Ge et al., 2006). The three most economically important *Zoysia* species are *Zoysia japonica* Steud., *Zoysia matrella* [L.] Merr. and *Zoysia pacifica* (Goudswaard) M. Hotta & Kuroki, and *Z. matrella* has the highest salt tolerance among them (Yamamoto et al., 2016). *Z. matrella* is a perennial warm-season turfgrass belonging to the family Gramineae, subfamily Chloridoideae, *Zoysia* willd (Tanaka et al., 2016a). Although the strong salt tolerance of *Z. matrella* is well known, the molecular mechanism is still unknown.

The current research on the salt tolerance of *Z. matrella* mainly focuses on the evaluation of salt tolerance and the physiological mechanism of salt tolerance. Salt tolerance evaluation shows that *Z. matrella* has strong salt tolerance (Marcum and Murdoch, 1994; Li et al., 2012). The physiological mechanism of salt tolerance showed that *Z. matrella* has large bicellular salt glands on the adaxial side and can secrete excessive Na^+^ from the salt glands of leaves to maintain a low Na^+^/K^+^ ratio to resist salt stress (Chen et al., 2009; Yamamoto et al., 2016). When exposed to salt stress, *Z. matrella* can also synthesize organic matter, such as betaine, glycine, proline, and soluble sugars, to adjust its osmotic potential to alleviate the osmotic stress caused by high salt concentrations (Li et al., 2012). Furthermore, an appropriate concentration of phosphorus under salt stress can promote root growth, increase Na^+^ secretion in leaves and inhibit Na^+^ transportation from roots to leaves, which is beneficial to the salt resistance of *Z. matrella* (Jiang et al., 2013).

Currently, few molecular mechanism studies on the salt tolerance of *Z. matrella* have been reported. A type I vacuolar H^+^-pyrophosphatase (VP) gene, *ZmVP1*, was isolated in *Z. matrella*, and overexpressing this gene in *Arabidopsis thaliana* can promote plant growth with more Na^+^ and K^+^ in the leaves and higher activities of V-ATPase and V-PPase under salt stress (Chen et al., 2015a). Chen et al. (2015b) constructed a high-quality full-length cDNA expression library in yeast using a Gateway-compatible vector system and screened 16 candidate salt-tolerant genes in *Z. matrella* (Chen et al., 2015b). Yeast validation experiments show that these 16 candidate salt-tolerant genes can improve the salt tolerance of yeast and exhibit different transcription levels under salt stress (Chen et al., 2015b). However, the molecular mechanism of some of these candidate salt-tolerant genes in *Z. matrella*, including a protein disulfide isomerase ZmPDI (NCBI accession number: KM265179), is very limited.

Protein disulfide isomerase (PDI) is a member of the thioredoxin subfamily of redox proteins and has thiol-disulfide oxidoreductase, disulfide isomerase and redox-dependent chaperone catalytic activities (Ali Khan and Mutus, 2014). PDI is mainly distributed throughout the lumen of the endoplasmic reticulum, cell surfaces and cytosol (Ali Khan and Mutus, 2014). It promotes the correct folding of proteins by catalyzing the formation of disulfide bonds and the rearrangement of mismatched disulfide bonds (Okumura et al., 2021). The functions of *PDI* in mammals have been extensively studied, while the functions in plants have less research. Existing studies suggest that, in *Arabidopsis*, *AtPDI11* has been demonstrated to exhibit oxidoreductase activity *in vitro* (Fan et al., 2018). The *AtPDI1* gene has an anti-stress function in *Arabidopsis*, and overexpressing *AtPDI1* can increase the abiotic stress tolerance of seedlings with a higher germination ratio and longer root length (Zhang et al., 2018). Overexpression of the protein disulfide isomerase *AtCYO1* has a negative effect on the initiation of chlorophyll degradation and proteolysis to maintain the functions of chloroplasts (Tominaga et al., 2018). However, studies on the regulatory mechanism of plant salt tolerance by the *PDI* gene have not been reported.

Previous studies showed that the heterologous expression of *ZmPDI* can significantly improve the salt tolerance of yeast, and the expression of *ZmPDI* is significantly upregulated after salt stress in *Z. matrella* (Chen et al., 2015b). Furthermore, *ZmPDI*-overexpressing transgenic *Z. matrella* plants have been successfully obtained (Wang K. et al., 2020). In order to further explore the regulation mechanism of *ZmPDI* in salt tolerance of *Z. matrella*, we evaluated the salt tolerance of *ZmPDI*-overexpressing transgenic *Z. matrella* plants, analyzed the gene ontology (GO) and Kyoto Encyclopedia of Genes and Genomes (KEGG) pathways that *ZmPDI* gene may affect after salt treatment and selected key differentially expressed genes (DEGs) that may be regulated by *ZmPDI* through combined transcriptome and proteome analysis. This study provides new insight into the regulatory mechanism of *ZmPDI* in *Z. matrella* for resistance to salt stress and facilitates the use of molecular breeding to improve the salt tolerance of grasses.

## Materials and methods

### Plant materials and treatments

The test materials were five *OX-ZmPDI* transgenic lines (Lines 4, 5, 7, 11, and 15) (Wang K. et al., 2020) and wild-type plants of *Z. matrella*, which are conserved by the Grass Research Center of Institute of Botany, Jiangsu Province and Chinese Academy of Sciences. The callus of *Z. matrella* was supported by Prof. Mingliang Chai from Zhejiang University (China).

Five *OX-ZmPDI* transgenic lines and wild-type plants were cultured in porous PVC tubes (height 40 cm, diameter 10 cm) filled with sand, and each material was cultured in 8 tubes. Water was poured into each tube material once a day, and Hoagland nutrient solution was poured and trimmed once every 5 days. When the growth was consistent, four tubes of each material were treated with NaCl solution, and the other four tubes were treated with water as the control. During salt treatment, 100 mM NaCl was used as the gradient to increase the concentration once every 3 days for progressive treatment with 400 mL each time. When the salt concentration reached 400 mM, the concentration no longer increased. Afterward, the material was treated with 400 mM NaCl every 5 days. The control and salt treatments were irrigated at the same time with 400 mL of water. Phenotypes of treated plants were observed after 30 days, and corresponding physicochemical indices of roots and leaves were determined.

The five *OX-ZmPDI* transgenic lines showed similar phenotypes and corresponding physicochemical index determination results, and transgenic Line 4 was selected for the next treatment and sampling. The stolons with ten nodes of *OX-ZmPDI* transgenic Line 4 and wild-type plants were selected and cut into small pieces with 2 nodes, and 40 small pieces of each material were planted. The hydroponic experiment was performed in the greenhouse. The hydroponic solution was 1/2 Hoagland nutrient solution, which was replaced once a week. When the growth of plants was consistent, 400 mM NaCl solution was added to the 1/2 Hoagland nutrient solution for salt treatment.

### Physiological index measurement

The physicochemical indices of *OX-ZmPDI* transgenic lines and wild-type plants in porous PVC tubes were determined after salt treatment for 30 days. The chlorophyll contents of leaves were determined by the acetone extraction method. The free proline contents of roots were determined by the sulfosalicylic acid method. The soluble sugar contents of roots were determined by the anthrone colorimetric method. The SOD activity of roots was determined by a total superoxide dismutase kit (hydroxylamine method) (Nanjing Jiancheng Bioengineering Institute, Nanjing, China). The Na^+^ and K^+^ contents in roots and leaves were determined according to the method of Wang J. et al. (2020). All data were analyzed for variance by SPSS Statistics v.26.0 (SPSS Inc., Chicago, IL, United States) and plotted by GraphPad Prism v.8 (GraphPad Software, San Diego, CA, United States).

### RNA-seq

The roots of *OX-ZmPDI* transgenic Line 4 and wild-type plants were sampled at 0 and 24 h after salt treatment, and three biological replicates were obtained for each sample at each time point. All samples were frozen in liquid nitrogen and stored at −80°C. Total RNA was extracted using a TRIzol reagent kit (Invitrogen, Carlsbad, CA, United States) according to the manufacturer’s protocol. A total of 12 cDNA libraries were constructed and sequenced by Gene *Denovo* Biotechnology Co., (Guangzhou, China) using the Illumina HiSeq2500 platform. The datasets are available in the NCBI repository http://www.ncbi.nlm.nih.gov/bioproject/PRJNA848971. *De novo* assembly of the *Z. matrella* transcriptome was accomplished *via* Hisat2 (v2.0.5) (Kim et al., 2015) using the *Z. japonica* genome as a reference (Tanaka et al., 2016b). The mapping reads of each sample were assembled using StringTie v1.3.1 (Pertea et al., 2015), and their expression abundance and variations were calculated by FPKM (fragment per kilobase of transcript per million mapped reads) values (Mortazavi et al., 2008). RNA differential expression analysis was performed by DESeq2 (Love et al., 2014) software and edgeR R package (3.18.1) (Robinson et al., 2010), which were used to analyze significant differences between two groups (determined as a *p*-value < 0.05 and absolute fold change ≥ 2). The DEGs were selected with a |log_2_FC| > 1 and false discovery rate (FDR) < 0.05.

### Data-independent acquisition protein quantification

For data-independent acquisition (DIA) proteomics analysis, proteins from the root samples (the same as RNA-seqsea) were collected from OX-ZmPDI transgenic Line 4 and wild-type plants at 0 and 24 h after salt treatment. Total proteins were extracted using the cold acetone method. After grinding, each sample was dissolved in 2 mL of lysis buffer (8 M urea, 2% SDS, 1 × protease inhibitor cocktail), pyrolyzed by sonication on ice for 30 min and collected by centrifugation at 13,000 rpm for 30 min at 4°C. The supernatant was retained, and prechilled acetone was added to precipitate the proteins at −20°C overnight. After cleaning with acetone three times, the precipitants were dissolved in 8 M urea by sonication on ice. The protein concentration of each sample was determined by a BCA protein assay kit.

For each sample, 50 μg of protein was suspended in 50 μL of solution, and 1 μL of 1 M dithiothreitol (DTT) was added at 55°C for 1 h. Then, 5 μL of 20 mM indoacetamide was used to alkylate the proteins of each sample in the dark at 37°C for 1 h. Then, 300 μL of prechilled acetone was added to each sample to precipitate the proteins at −20°C overnight, and the proteins were washed twice with prechilled acetone. The proteins were resuspended with 50 mM ammonium bicarbonate and digested with sequence-grade modified trypsin (Promega, Madison, WI, United States) at a substrate/enzyme ratio of 50:1 (w/w) at 37°C for 16 h.

The spectral libraries were generated (Fang et al., 2021), and the spectrophotometer was set up to search the database of *Z. matrella*.^1^ After nano-HPLC–MS/MS analysis, the raw DIA data were acquired, processed and analyzed by Spectronaut X (Biognosys AG, Switzerland) with default parameters (Fang et al., 2021). After performing Student’s *t*-test, differentially expressed proteins (DEPs) were filtered with a *q*-value < 0.05 and |Fold change (FC)| > 1.5.

### Bioinformatics analysis

The correlation analysis of replicas was performed by R. Correlation to evaluate repeatability between three biological replicate samples. Principal component analysis (PCA) was performed with the R package gmodels^2^ to reveal the relationship of all the samples. The correlation analysis of RNA-seq and proteome was performed by R (version 3.5.1), and a nine-quadrant map was drawn based on changes in the expression of the gene in the transcriptome and proteome. All DEP and DEG functions were annotated in the GO database, KEGG database and Clusters of Orthologous Groups of proteins (COG/KOG) database. The significantly enriched GO functions and KEGG pathways were examined within DEGs and DEPs with FDR ≤ 0.05 and *q*-value ≤ 0.05, respectively.

### Quantitative RT-PCR validation

Twelve DEGs were randomly selected from the gene list in Table 1 for qRT–PCR validation. The primers were designed by Primer Premier 5.0 software and are listed in Supplementary Table 10. The *ZjActin* (GenBank: GU290545.1) gene was used as a housekeeping gene. Each sample was analyzed with three biological replicates, and qRT–PCR assays were carried out as described by Xie et al. (2015).

**TABLE 1:** Selected differentially expressed genes (DEGs) from transcriptome and proteome association analysis.

GeneID	RNA-seq			Proteome			Gene name	Description
	FC1	FC2	log_2_	FC3	FC4	log_2_		
	(WT24h/0h)	(ZmPDI24h/0h)	(FC2/FC1)	(WT24h/0h)	(ZmPDI24h/0h)	(FC4/FC3)		
Zjn_sc00047.1.g03800.1.sm.mk	5.10	11.14	1.13	1.77	1.05	−0.75	\	Hypothetical protein
Zjn_sc00022.1.g04760.1.sm.mk	0.23	0.21	−0.10	1.02	0.22	−2.23	\	WEB family protein At5g16730, chloroplastic
Zjn_sc00015.1.g05160.1.sm.mkhc	0.12	0.09	−0.46	0.98	0.32	−1.62	\	\
**Zjn_sc00089.1.g00090.1.am.mk**	**0.36**	**0.17**	−**1.11**	**1.00**	**0.42**	−**1.25**	**4CL1**	**4-coumarate-CoA ligase 1**
Zjn_sc00109.1.g00965.1.br	6.83	16.91	1.31	1.74	1.93	0.15	BBE	Berberine bridge enzyme-like Cyn d 4
Zjn_sc00018.1.g01620.1.sm.mkhc	0.16	0.35	1.12	0.70	0.51	−0.46	CCR1	Cinnamoyl-CoA reductase 2
Zjn_sc00090.1.g01530.1.sm.mkhc	2.90	3.10	0.10	0.84	2.93	1.80	CEQORH	Chloroplast envelope quinone oxidoreductase homolog
Zjn_sc00109.1.g00880.1.sm.mk	21.70	22.30	0.04	1.85	0.90	−1.04	CHT2	Chitinase 2-like
Zjn_sc00102.1.g00830.1.am.mk	38.47	16.84	−1.19	2.67	0.80	−1.74	CHT8	Chitinase 8
Zjn_sc00045.1.g00590.1.sm.mk	0.14	0.01	−3.84	0.80	0.56	−0.51	CYP74A4	Allene oxide synthase 4
Zjn_sc00013.1.g09290.1.am.mkhc	0.71	3.73	2.40	0.77	4.19	2.45	ephA	Epoxide hydrolase A-like
Zjn_sc00012.1.g02790.1.am.mkhc	0.31	0.40	0.34	0.86	0.42	−1.04	FPS	Farnesyl pyrophosphate synthase
Zjn_sc00049.1.g01050.1.am.mk	0.23	0.11	−1.06	0.69	0.39	−0.85	GLIP	GDSL esterase/lipase
Zjn_sc00048.1.g00650.1.sm.mkhc	0.43	0.42	−0.05	0.48	1.29	1.42	GSH2	Glutathione synthetase, chloroplastic-like isoform X1
Zjn_sc00003.1.g11520.1.sm.mk	0.33	0.21	−0.62	0.52	1.20	1.19	H2AV	Probable histone H2A variant 1
Zjn_sc00003.1.g07680.1.sm.mkhc	2.91	6.27	1.11	0.98	2.00	1.03	HB2	Non-symbiotic hemoglobin
Zjn_sc00011.1.g02850.1.sm.mkhc	0.22	0.09	−1.25	1.10	0.53	−1.06	LAMP1	Probable glutamate carboxypeptidase LAMP1
Zjn_sc00026.1.g00700.1.sm.mk	104.07	51.72	−1.01	3.53	1.63	−1.11	LEA14-A	Late embryogenesis abundant protein Lea14-A-like
Zjn_sc00075.1.g00370.1.sm.mk	0.43	0.59	0.45	0.54	1.11	1.03	LOX5	Linoleate 9S-lipoxygenase 5
Zjn_sc00034.1.g03480.1.am.mk	0.11	0.13	0.19	0.70	0.32	−1.16	LTP	Protease inhibitor/seed storage/LTP family protein precursor
Zjn_sc00011.1.g06270.1.am.mk	\	0.04	\	0.62	0.15	−2.06	MEE55	Serinc-domain containing serine and sphingolipid biosynthesis protein
Zjn_sc00155.1.g00490.1.am.mk	0.05	0.22	2.12	0.42	0.31	−0.43	NRT2.1	High-affinity nitrate transporter 2.1-like
Zjn_sc00152.1.g00350.1.sm.mk	0.05	0.15	1.43	0.43	0.46	0.08	NRT2.1	High-affinity nitrate transporter 2.1-like
Zjn_sc00052.1.g00190.1.sm.mkhc	0.03	0.09	1.37	0.95	0.57	−0.75	OMT2	O-methyltransferase 2
**Zjn_sc00005.1.g03630.1.sm.mkhc**	**2.70**	**3.06**	**0.18**	**0.98**	**1.98**	**1.02**	**PFK4**	**ATP-dependent 6-phosphofructokinase 4 chloroplastic**
**Zjn_sc00009.1.g08860.1.sm.mkhc**	**0.15**	**0.06**	−**1.28**	**0.88**	**0.66**	−**0.42**	**PLDα2**	**Phospholipase D alpha 2**
Zjn_sc00026.1.g02680.1.am.mk	0.45	0.08	−2.46	1.40	0.38	−1.89	PNC1	Cationic peroxidase 1-like
Zjn_sc00143.1.g00490.1.sm.mkhc	1.69	3.15	0.90	0.84	1.83	1.11	pro-resilin	Pro-resilin precursor
**Zjn_sc00093.1.g00470.1.sm.mkhc**	**0.06**	**0.03**	−**1.13**	**1.29**	**0.59**	−**1.12**	**PXG4**	**Peroxygenase 4**
Zjn_sc00207.1.g00180.1.cf.mkhc	0.29	0.23	−0.33	0.29	0.69	1.25	RPM1	Disease resistance protein RPM1
Zjn_sc00152.1.g00230.1.sm.mk	0.48	0.45	−0.10	0.33	1.25	1.92	RPPR5	Pentatricopeptide repeat-containing protein At2g37230
Zjn_sc00004.1.g14080.1.sm.mkhc	0.09	0.08	−0.08	0.22	0.45	1.06	STY8	Serine/threonine-protein kinase STY8
Zjn_sc00022.1.g06510.1.sm.mkhc	0.19	0.24	0.29	0.99	0.47	−1.06	TIP2-1	Aquaporin TIP2-1
Zjn_sc00007.1.g10230.1.sm.mkhc	0.28	0.35	0.32	0.99	0.47	−1.06	TIP2-1	Aquaporin TIP2-1
**Zjn_sc00009.1.g09140.1.sm.mk**	**0.20**	**0.07**	−**1.55**	**0.77**	**0.48**	−**0.69**	**TUBB2**	**Tubulin beta-2 chain**
Zjn_sc00096.1.g01880.1.am.mk	54.13	86.21	0.67	4.61	2.25	−1.03	XIP2	Xylanase inhibitor protein 2-like

## Results

### Salt tolerance evaluation of *OX-ZmPDI* transgenic plants of *Zoysia matrella*

**FIGURE 1:**
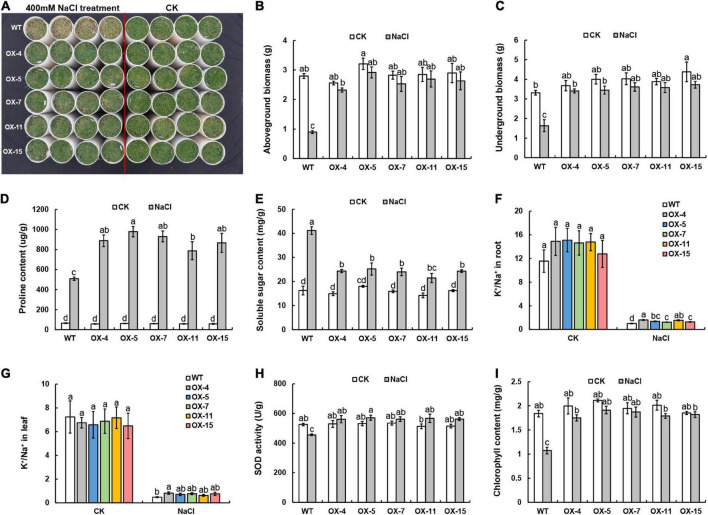
Phenotypic and physiological responses to salt stress in *OX-ZmPDI transgenic plants of Z. matrella.* **(A)** *OX-ZmPDI* transgenic lines and wild-type (WT) plants were exposed to 400 mM NaCl and control (CK) treatments for 30 days. **(B)** The aboveground biomasses of *OX-ZmPDI* transgenic and wild-type plants after NaCl and CK treatments for 30 days. **(C)** The underground biomasses of *OX-ZmPDI* transgenic and wild-type plants after NaCl and CK treatments for 30 days. **(D)** The proline contents of *OX-ZmPDI* transgenic and wild-type plants after NaCl and CK treatments for 30 days. **(E)** The soluble sugar contents of *OX-ZmPDI* transgenic and wild-type plants after NaCl and CK treatments for 30 days. **(F)** The K^+^/Na^+^ ratio in *OX-ZmPDI* transgenic and wild-type plant roots after CK and NaCl treatments. **(G)** The K^+^/Na^+^ ratio in *OX-ZmPDI* transgenic and wild-type plant leaves after CK and NaCl treatments. **(H)** The chlorophyll contents of *OX-ZmPDI* transgenic and wild-type plants after NaCl and CK treatments for 30 days. **(I)** The SOD activity of *OX-ZmPDI* transgenic and wild-type plants after NaCl and CK treatments for 30 days. Values are presented as the mean ± SE. Letters above the bars indicate significant differences between the respective values (*p* < 0.05).

To evaluate the salt tolerance of *OX-ZmPDI* transgenic lines, 400 mM NaCl solution was applied for 30 days to treat the *OX-ZmPDI* transgenic lines and wild-type plants. After salt treatment, the wild-type plants were obviously withered and yellowed, while the *OX-ZmPDI* transgenic lines all maintained normal growth, which was not significantly different from the control (CK) treatment (Figure 1A). In the wild-type plants, the dry weights of the aboveground and underground biomasses of the salt-treated plants were significantly lower than those of the CK plants (Figures 1B,C). However, the dry weights of the aboveground and underground biomasses of the *OX-ZmPDI* transgenic lines were not significantly different between the salt and CK treatments (Figures 1B,C).

After salt treatment, the contents of the osmotic adjustment substances, proline and sugars, were detected in *OX-ZmPDI* transgenic lines and wild-type plants. The proline contents of wild-type plants increased after salt treatment, and the proline contents of *OX-ZmPDI* transgenic lines were much higher than those of wild-type plants (Figure 1D). However, the soluble sugar contents of *Z. matrella* plants all increased after salt treatment, and the degree of increase in *OX-ZmPDI* transgenic lines was significantly lower than that in wild-type plants (Figure 1E). The K^+^/Na^+^ ratio is also an important index with which to evaluate the salt tolerance of plants. In the CK treatment, the K^+^/Na^+^ ratios in roots and leaves were not significantly different between the wild-type and *OX-ZmPDI* transgenic plants (Figures 1F,G). However, after treatment with 400 mM NaCl, the K^+^/Na^+^ ratios of *OX-ZmPDI* transgenic lines in roots were all obviously higher than those of wild-type plants, especially *OX-ZmPDI* transgenic line OX-4 (Figure 1F). Meanwhile, the K^+^/Na^+^ ratios in the leaves of *OX-ZmPDI* transgenic line OX-4 were significantly higher than those of the wild-type plants (Figure 1G). Furthermore, chlorophyll contents and superoxide dismutase (SOD) activity are also important for assessing salt tolerance. In this study, the chlorophyll content of wild-type plants was obviously lower than that of the CK treatment, while the chlorophyll contents of *OX-ZmPDI* transgenic lines showed no significant difference compared with the CK treatment (Figure 1H). After salt treatment, the SOD activity of wild-type plants showed an obviously downward trend, while that of *OX-ZmPDI* transgenic lines showed no significant difference compared with the CK treatment (Figure 1I).

### Transcriptome and proteome sequencing of *OX-ZmPDI* transgenic plants

Previous studies show that roots may make significant contributions to the salt tolerance in zoysiagrass (Wang J. et al., 2020). In the preliminary physiological index measurement, the K^+^/Na^+^ ratios were measured both in roots and leaves. The results showed that the K^+^/Na^+^ ratios in roots of all *OX-ZmPDI* transgenic lines were obviously higher than those of wild-type plants, while only the K^+^/Na^+^ ratio in leaves of *OX-ZmPDI* transgenic line OX-4 was significantly higher than wild-type plants (Figures 1F,G). Therefore, in order to analyze the molecular mechanism by which the *ZmPDI* gene enhances the salt tolerance of *Z. matrella*, root samples of the *OX-ZmPDI* transgenic line OX-4 and wild-type plants were collected at 0 and 24 h after treatment with 400 mM NaCl for transcriptome and proteome sequencing. For RNA-seq, an average of 43.24 million raw reads were obtained, and 97.98% of these reads were confirmed as clean reads (Supplementary Table 1). A total of 69.05–75.64, 15.10−20.39, and 8.65–10.38% of the reads were mapped to exons, introns and intergenic regions in the reference genome, respectively (Supplementary Table 2). In total, 35,197 genes were identified by the RNA-seq assays, including 33,660 unigenes and 1,537 novel genes.

DIA technology was used to perform the comparative analysis of *OX-ZmPDI* transgenic line OX-4 and wild-type plants. More than half of the peptides had lengths between 7 and 18 amino acids (Supplementary Figure 1A). After filtering with FDR ≤ 0.05, 36,076 peptides and 6,842 proteins were obtained, and 5,040 (73.66%) proteins had more than one peptide (Supplementary Figures 1B,C). Among the total 6,842 proteins, 6,370 (93.10%) were annotated in at least one of the GO, KEGG and KOG protein libraries (Supplementary Figure 1D). Transcription factor analysis showed that most proteins were in the *bZIP* transcription factor family, followed by the *C3H* and Trihelix transcription factor families (Supplementary Figure 1E).

**FIGURE 2:**
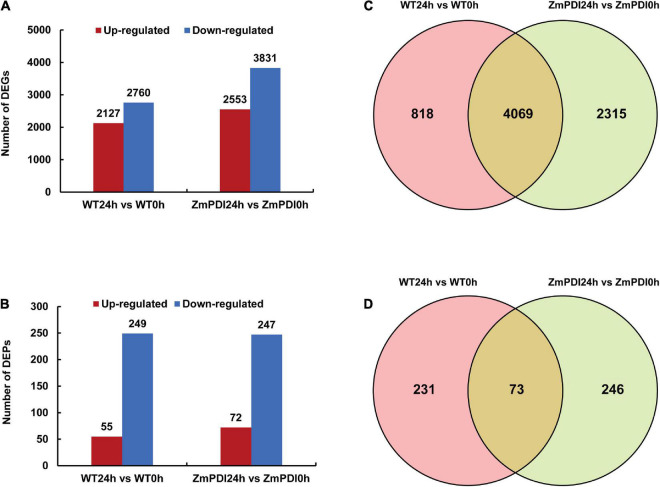
Salt tolerance-related transcriptome and proteome expression profiles of *OX-ZmPDI* transgenic and WT plants of *Z. matrella.* **(A)** The number of up- and downregulated DEGs in *OX-ZmPDI* transgenic (ZmPDI24h vs. ZmPDI0h) and wild-type plants (WT24h vs. WT0h). **(B)** The number of up- and downregulated DEPs in *OX-ZmPDI* transgenic (ZmPDI24h vs. ZmPDI0h) and wild-type plants (WT24h vs. WT0h). **(C)** Venn diagram of the number of DEGs in the *OX-ZmPDI* transgenic and wild-type plants after salt treatment. **(D)** Venn diagram of the number of DEPs in the *OX-ZmPDI* transgenic and wild-type plants after salt treatment.

The correlation values of biological repeat samples for RNA-seq and the proteome were all above 0.866 (Supplementary Figures 2A,B). A total of 4,887, 11, 9, and 6,384 DEGs were identified in the WT24h vs. WT0h, ZmPDI0h vs. WT0h, ZmPDI24h vs. WT24h and ZmPDI24h vs. ZmPDI0h comparisons, respectively (Supplementary Figure 2C). Because the numbers of DEGs in the ZmPDI0h vs. WT0h and ZmPDI24h vs. WT24h comparisons were very low, we chose WT24h vs. WT0h and ZmPDI24h vs. ZmPDI0h comparisons to continue the further analysis, and found that the downregulation of DEGs in these two comparisons was greater than the upregulation of DEGs (Figure 2A). For proteome sequencing, 304 and 319 DEPs were identified in the WT24h vs. WT0h and ZmPDI24h vs. ZmPDI0h comparisons, respectively, and the number of downregulated DEPs was 4.52 and 3.43 times that of upregulated DEPs in these two comparisons, respectively (Figure 2B). Venn diagrams were constructed and showed that the WT24h vs. WT0h and ZmPDI24h vs. ZmPDI0h comparisons had 4,069 (56.50%) DEGs in common, while only 73 (13.27%) common DEPs were common (Figures 2C,D).

### Gene ontology and Kyoto Encyclopedia of Genes and Genomes pathway analysis of differentially expressed genes and differentially expressed proteins

**FIGURE 3:**
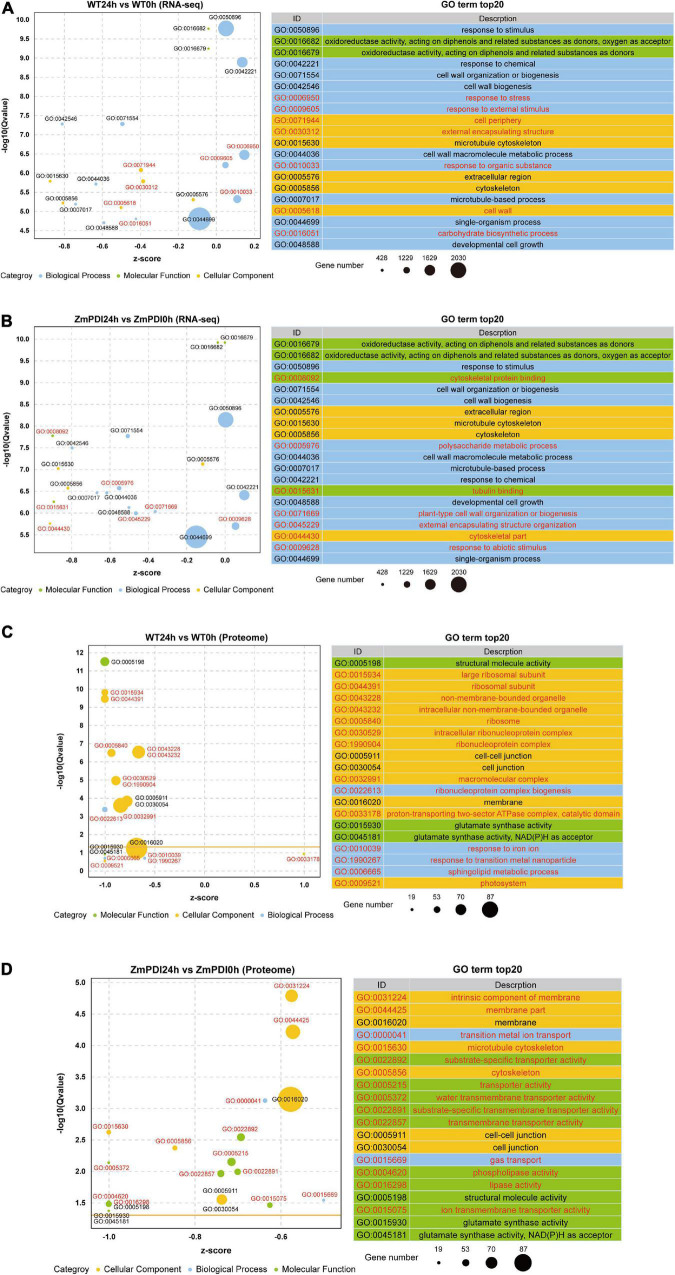
Top 20 enriched GO terms of DEGs and DEPs in *OX-ZmPDI* transgenic and WT plants of *Z. matrella*. **(A)** Top 20 enriched GO terms of DEGs in the WT24h vs. WT0h comparison. **(B)** Top 20 enriched GO terms of DEGs in the ZmPDI24h vs. ZmPDI0h comparison. **(C)** Top 20 enriched GO terms of DEPs in the WT24h vs. WT0h comparison. **(D)** Top 20 enriched GO terms of DEPs in the ZmPDI24h vs. ZmPDI0h comparison. The *z*-score value represents the ratio of the difference between the numbers of upregulated and downregulated genes to the total number of differentially expressed genes. The yellow line represents the threshold of *Q*-value = 0.05.

To compare the differences in salt tolerance regulation mechanisms between wild-type and *OX-ZmPDI* transgenic plants, the functional characterization of DEGs and DEPs was classified by GO enrichment analysis. For RNA-seq, the top 20 enriched GO terms were selected in the WT24h vs. WT0h and ZmPDI24h vs. ZmPDI0h comparisons (Supplementary Table 3). A total of 13 GO terms were shared by both the WT24h vs. WT0h and ZmPDI24h vs. ZmPDI0h comparisons, while each comparison had seven unique GO terms (Figures 3A,B). In the ZmPDI24h vs. ZmPDI0h comparison, the unique GO terms included “cytoskeletal protein binding,” “polysaccharide metabolic process,” “tubulin binding,” “plant-type cell wall organization or biogenesis,” “external encapsulating structure organization,” “cytoskeletal part” and “response to abiotic stimulus,” and multiple terms were mainly related to the cytoskeleton (Figure 3B). Furthermore, except for “response to abiotic stimulus,” these unique GO terms in the ZmPDI24h vs. ZmPDI0h comparison had more downregulated DEGs (Figure 3B).

For proteome sequencing, the top 20 enriched GO terms were selected in the WT24h vs. WT0h and ZmPDI24h vs. ZmPDI0h comparisons, and only six GO terms were shared by both comparisons (Figures 3C,D and Supplementary Table 4). The WT24h vs. WT0h and ZmPDI24h vs. ZmPDI0h comparisons had 14 unique GO terms, and eight unique GO terms in the ZmPDI24h vs. ZmPDI0h comparison were related to transport or transport activity, including “transition metal ion transport,” “substrate-specific transporter activity,” “transporter activity,” “water transmembrane transporter activity,” “substrate-specific transmembrane transporter activity,” “transmembrane transporter activity,” “gas transport” and “ion transmembrane transporter activity” (Figures 3C,D). Comparing the top 20 enriched GO terms in the ZmPDI24h vs. ZmPDI0h comparison between RNA-seq and proteome sequencing, “microtubule cytoskeleton” and “cytoskeleton” terms were shared, and the DEGs in these two terms were mainly downregulated genes (Figures 3B,D).

**FIGURE 4:**
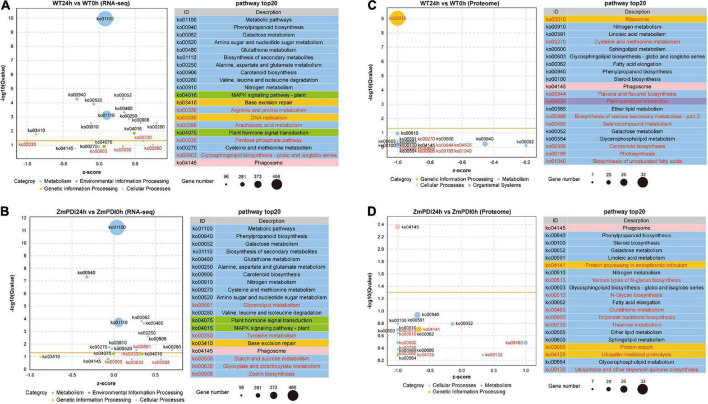
Top 20 enriched GO terms of DEGs and DEPs in *OX-ZmPDI* transgenic and WT plants of *Z. matrella*. **(A)** Top 20 enriched KEGG pathways of DEGs in the WT24h vs. WT0h comparison. **(B)** Top 20 enriched KEGG pathways of DEGs in the ZmPDI24h vs. ZmPDI0h comparison. **(C)** Top 20 enriched KEGG pathways of DEPs in the WT24h vs. WT0h comparison. **(D)** Top 20 enriched KEGG pathways of DEPs in the ZmPDI24h vs. ZmPDI0h comparison. The *z*-score value represents the ratio of the difference between the numbers of upregulated and downregulated genes to the total number of differentially expressed genes. The yellow line represents the threshold of *Q*-value = 0.05.

The KEGG pathway analysis of DEGs for RNA-seq showed that 15 pathways were shared by both the WT24h vs. WT0h and ZmPDI24h vs. ZmPDI0h comparisons, and each comparison had five unique pathways (Figures 4A,B and Supplementary Table 5). In the ZmPDI24h vs. ZmPDI0h comparison, the five unique pathways included “Glycerolipid metabolism,” “Tyrosine metabolism,” “Starch and sucrose metabolism,” “Glyoxylate and dicarboxylate metabolism” and “Zeatin biosynthesis” (Figure 4B). Except for “starch and sucrose metabolism,” the other four unique pathways had more upregulated DEGs (Figure 4B). KEGG pathway analysis of DEPs for proteome sequencing showed that the top 20 pathways were selected in the WT24h vs. WT0h and ZmPDI24h vs. ZmPDI0h comparisons, and each comparison had nine unique pathways (Figures 4C,D and Supplementary Table 6). In the ZmPDI24h vs. ZmPDI0h comparison, multiple unique pathways were related to proteins, including “Protein processing in endoplasmic reticulum,” “Protein export” and “Ubiquitin mediated proteolysis,” and DEPs in these pathways were all downregulated (Figure 4D). Comparing the pathways in the ZmPDI24h vs. ZmPDI0h comparison, “phenylpropanoid biosynthesis,” “galactose metabolism,” “glutathione metabolism,” “nitrogen metabolism” and “phagosome” were shared between RNA-seq and proteome sequencing (Figures 4B,D).

### Transcriptome and proteome association analysis

To screen the DEGs with the same expression changes in the transcriptome and proteome, we performed an association analysis. In the WT24h vs. WT0h comparison, assessment of the number of genes revealed that there were 104 DEGs upregulated in the transcriptome and proteome, including 78 DEGs that did not meet the *P*-value threshold for significance (Supplementary Table 7). Meanwhile, 276 DEGs were downregulated in the transcriptome and proteome, including 240 DEGs that did not meet the *P*-value threshold (Supplementary Table 7). In the ZmPDI24h vs. ZmPDI0h comparison, the analysis of the number of genes revealed that there were 125 DEGs upregulated in the transcriptome and proteome, with 97 DEGs not meeting the *P*-value threshold (Supplementary Table 7). At the same time, 342 DEGs (including 285 DEGs that did not meet the *P*-value) were downregulated in the transcriptome and proteome (Supplementary Table 7).

The nine quadrant diagrams showed that upregulated and downregulated DEGs in the transcriptome and proteome were in quadrants 3 and 7, respectively, and the DEGs meeting the *P*-value are marked by red dots (Supplementary Figures 2C,D). Then, GO enrichment and KEGG pathway analyses were performed with the DEGs that met the *P*-value in quadrants 3 and 7. The GO enrichment analysis showed that the “cytoskeleton” and “microtubule cytoskeleton” terms were also more significantly enriched in the ZmPDI24h vs. ZmPDI0h comparison than in the WT24h vs. WT0h comparison (Supplementary Figures 3A,B and Supplementary Table 8). Comparing the KEGG pathways in the WT24h vs. WT0h comparison, seven unique metabolic pathways were enriched in the ZmPDI24h vs. ZmPDI0h comparison, including “ether lipid metabolism,” “stilbenoid, diarylheptanoid and gingerol biosynthesis,” “glycerophospholipid metabolism,” “cutin, suberine and wax biosynthesis,” “ubiquinone and other terpenoid-quinone biosynthesis,” “pentose phosphate pathway” and “glycolysis/gluconeogenesis” (Supplementary Figures 3C,D and Supplementary Table 9).

### Differentially expressed gene identification and verification

In total, 119 DEGs that met the *P*-value threshold in quadrants 3 and 7 were selected from the WT24h vs. WT0h and ZmPDI24h vs. ZmPDI0h comparisons (Supplementary Figure 4). Most of these DEGs had similar expression trends between the WT24h vs. WT0h and ZmPDI24h vs. ZmPDI0h comparisons, and the protein levels of most DEGs were consistent with their RNA levels (Supplementary Figure 4). The number of DEGs in the *OX-ZmPDI* transgenic plants was very low compared with the wild-type plants either at the time point of salt treatment at 0h or 24h (Supplementary Figure 2C), which indicating that the degree of change in gene expression induced by salt stress in wild-type and *OX-ZmPDI* transgenic plants may be more important. Therefore, we set a selected condition of |log_2_[FC(ZmPDI24h/ZmPDI0h)/FC(WT24h/WT0h)]| > 1 and screened 36 DEGs significantly different between WT24h vs. WT0h and ZmPDI24h vs. ZmPDI0h comparisons at the RNA or proteome levels (Table 1).

**FIGURE 5:**
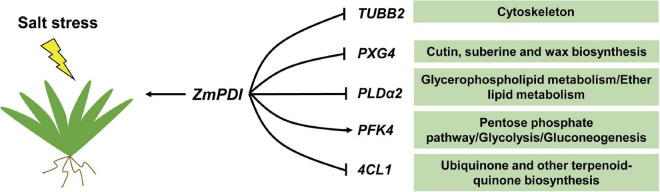
A predictive regulatory model of *ZmPDI* enhancing salt tolerance in *Z. matrella*. The contents in the green boxes are the GO term or KEGG pathways of the genes on the left belong.

Previous GO and KEGG pathway analyses in quadrants 3 and 7 showed that “cytoskeleton,” “microtubule cytoskeleton,” “ether lipid metabolism,” “stilbenoid, diarylheptanoid and gingerol biosynthesis,” “glycerophospholipid metabolism,” “cutin, suberine and wax biosynthesis,” “ubiquinone and other terpenoid-quinone biosynthesis,” “pentose phosphate pathway” and “glycolysis/gluconeogenesis” are significantly enriched in the *OX-ZmPDI* transgenic plants (Supplementary Figure 3). According to these GO terms and KEGG pathways, five important DEGs (*TUBB2*, *PXG4*, *PLD*α*2*, *PFK4*, and *4CL1*) were selected from 36 DEGs, and they belonged to “cytoskeleton,” “cutin, suberine and wax biosynthesis,” “glycerophospholipid metabolism/ether lipid metabolism,” “pentose phosphate pathway/glycolysis/gluconeogenesis” and “ubiquinone and other terpenoid-quinone biosynthesis” respectively (Figure 5). Comparing to wild-type plants, the expression and protein levels of *TUBB2*, *PXG4*, *PLD*α*2*, and *4CL1* were downregulated and the levels of *PFK4* were upregulated in the *OX-ZmPDI* transgenic plants under salt stress (Figure 5). These results indicated that the *ZmPDI* gene may enhance the salt tolerance of zoysiagrass by affecting the expression level or protein level of these genes. To confirm the reliability of RNA-seq, 12 genes selected from 36 DEGs (Table 1) were validated using qRT–PCR, and the results were largely consistent with the RNA-seq data (Supplementary Figure 5).

## Discussion

### Overexpression of *ZmPDI* can enhance the salt tolerance of *Zoysia matrella*

Previous studies have shown that heterologous expression of *ZmPDI* enhances the salt tolerance of yeast (Chen et al., 2015b), but whether it has the same function in *Z. matrella* remains to be verified. In this study, homologous expression of *ZmPDI* in *Z. matrella* significantly improved salt tolerance while maintaining normal growth and aboveground and underground biomasses (Figures 1A–C). Under salinity stress, plant growth and development are inhibited mainly because of osmotic pressure disturbance, ionic imbalance and oxidative stress (Zhao et al., 2020). Plants regulate osmotic pressure mainly through two pathways, namely, the synthesis of organic osmolytes and improved absorption of inorganic ions (Zhao et al., 2020). Proline and sugars are organic osmolytes, and proline plays a dominant role in regulating osmotic pressure under salt stress (Zhao et al., 2020). In the *OX-ZmPDI* transgenic lines, the proline contents were significantly higher than those in the wild-type plants, while the soluble sugar contents were significantly lower (Figures 1D,E). These results indicated that *OX-ZmPDI* transgenic plants may synthesize more proline and activate glucose metabolism to adjust osmotic pressure under salinity stress.

Many enzymes involved in primary metabolism are present in the cytosolic compartment, and many of them are controlled by K^+^ (Zhao et al., 2020). However, Na^+^ is very similar to K^+^ and usually replaces K^+^ to involve those enzymatic reactions with much less efficiency (Zhao et al., 2020). Therefore, a high concentration of Na^+^ can disrupt metabolism and potentially kill the plant (Zhao et al., 2020). In *OX-ZmPDI* transgenic lines, the K^+^/Na^+^ ratios in roots were all significantly higher than those in wild-type plants after salt treatment (Figure 1F), indicating that *ZmPDI* can help plants maintain higher K^+^/Na^+^ ratios to resist Na^+^ damage. Reactive oxygen species (ROS) are rapidly induced by salinity stress and are mainly produced in the apoplast, chloroplasts, mitochondria and peroxisomes (Zhao et al., 2020). SOD can scavenge excess ROS to enhance the salt tolerance of plants (Zhao et al., 2020). Under salt treatment, overexpression of the *ZmPDI* gene significantly maintained normal SOD activity and chlorophyll content in *Z. matrella* (Figures 1H,I), indicating that normal SOD activity in chlorophyll may catalyze the scavenging of ROS and maintain the normal function of chloroplasts. These results proved that *ZmPDI* can enhance the salt tolerance of plants by maintaining osmotic pressure and ionic balance and reducing oxidative stress.

### *ZmPDI* may enhance the salt tolerance of *Zoysia matrella* by regulating *TUBB2*, *PXG4*, *PLD*α*2*, *PFK4*, and *4CL1*

To understand the difference in salt tolerance responses between wild-type and *OX-ZmPDI* transgenic plants, an association analysis of transcriptome and proteome sequencing was performed. The GO enrichment and KEGG pathway analyses of 119 DEGs that met the *P*-value threshold in quadrants 3 and 7 showed that *ZmPDI* may enhance the salt tolerance of *Z. matrella* in five aspects, namely, “cytoskeleton,” “cutin, suberine and wax biosynthesis,” “glycerophospholipid metabolism/ether lipid metabolism,” “pentose phosphate pathway/glycolysis/gluconeogenesis” and “ubiquinone and other terpenoid-quinone biosynthesis” (Figure 5). The plant cytoskeleton is mainly composed of microtubules (MTs), microfilaments (MFs) and MT/MF-interacting proteins, and its dynamic organizational changes can enhance plant tolerance through various intracellular activities, including cell morphogenesis and cell signal transduction (Yang and Guo, 2018; Zhao et al., 2021). Salt stress induces the depolymerization and reorganization of MTs, and its destabilization can enhance the salt tolerance of plants (Zhao et al., 2021). However, MF depolymerization is related to cell death under long-term salt stress, and its stabilization can increase the ability of plants to withstand salt stress (Yang and Guo, 2018; Zhao et al., 2021). A cytoskeletal structural component gene, *beta-2-tubulin* (*TUBB2*, *Zjn_sc00009.1.g09140.1.sm.mk*), was screened as a DEG and downregulated in *OX-ZmPDI* transgenic plants at both the transcriptome and proteome levels (Table 1). Tubulin is the major constituent of the cytoskeleton, which forms networks of microtubules (Chiolerio et al., 2020). These results indicated that *ZmPDI* may improve the salt tolerance of zoysiagrass by downregulating TUBB2 levels to affect the destabilization of MTs.

Epoxygenated fatty acids are components of cutin and suberin and act as defense molecules (Blee et al., 2012). The caleosin-type peroxygenases catalyze the epoxidation of unsaturated fatty acids in the presence of hydroperoxides to form epoxygenated fatty acids (Blee et al., 2012). *Peroxygenase 4* (*PXG4*, *Zjn_sc00093.1.g00470.1.sm.mkhc*) is a class II caleosin gene that encodes a calcium-binding peroxygenase involved in the degradation of storage lipids (Blee et al., 2012). In *Arabidopsis*, another name of the *PXG4* gene is *AtCLO4*, and previous studies have confirmed that *PXG4* participates in salt tolerance by acting as a negative regulator of abscisic acid (ABA) (Kim et al., 2011; Blee et al., 2012). During salt stress, the *clo4* mutants perform significantly less inhibition of lateral root formation (Rafeh, 2016). After salt treatments, the “cutin, suberine and wax biosynthesis” and *PXG4* genes were significantly enriched and screened in *OX-ZmPDI* transgenic plants (Figure 5). Compared with wild-type plants, the transcript and protein levels of *PXG4* in *OX-ZmPDI* transgenic plants were all decreased (Table 1). These results indicated that *ZmPDI* may inhibit *PXG4* expression to decrease this protein level, thereby maintaining lateral root formation to improve the salt tolerance of zoysiagrass.

Phospholipase D (PLD) is a phosphatidyl choline-hydrolyzing enzyme that can generate the lipid second messenger phosphatidic acid (PA) by hydrolyzing membrane phospholipids, and the expression of multiple PLD genes is increased by exposure to various stresses (Bargmann and Munnik, 2006; Ji et al., 2017). PLDs are involved in multiple cellular processes, including membrane composition and microtubule and cytoskeletal dynamics (Faraudo and Travesset, 2007; Zhang et al., 2017). The expression levels of some PLD genes are induced by salt stress, and *pld*α*1*, *pld*α*3*, and *pld*δ mutants show salt sensitivity (Hunter et al., 2019). Among them, the molecular mechanism by which *PLD*α*1* responds to salt stress has been studied in depth. PLDα1 interacts with mitogen-activated protein kinases 3 (MPK3) to enhance salt tolerance and ABA signaling (Vadovič et al., 2019). In addition, Cys-rich receptor-like kinase 2 (CRK2) acts as the downstream target protein of PLDα1 and enhances salt tolerance at the germination stage in *Arabidopsis* (Hunter et al., 2019). Previous studies have shown that *PLD*α*2* is highly induced by salt and drought stress in rice (Li et al., 2007). However, the transcript and protein levels of *PLD*α*2* in *OX-ZmPDI* transgenic and wild-type plants all decreased after salt treatments (Table 1). The protein alignment of *PLD*α*2* genes in *Z. matrella* and *Oryza sativa* showed that the amino acid sequences of ZmPLDα2 and OsPLDα2 have an obviously difference (Supplementary Figure 6A). These results indicated that *PLD*α*2* may play a different role in regulating the salt tolerance of zoysiagrass.

In the plant glycolytic pathway, ATP-dependent 6-phosphofructokinase (PFK) catalyzes the phosphorylation of fructose 6-phosphate (F6P), transforming it to fructose-1,6-bisphosphate (F16BP) (Zhong et al., 2016). In previous studies, the expression levels of *PFK* were significantly induced by salt stress in rice, cucumber and tomato (Zhong et al., 2016; Yuenyong et al., 2018; Feng et al., 2019). Soluble sugar is an organic osmolyte that can balance the vacuole solute potential, but the accumulation of sugar in leaves can inhibit photosynthesis (Zhong et al., 2016). In our studies, compared with wild-type plants, the transcript and protein levels of *PFK4* in *OX-ZmPDI* transgenic plants were all increased (Table 1). The soluble sugar contents were significantly lower and the chlorophyll contents maintained significant high levels in *OX-ZmPDI* transgenic plant leaves (Figures 1E,H). These results indicated that *ZmPDI* may increase *PFK4* expression to accelerate the conversion of F6P into F16BP, thereby enhancing the glycolysis pathway to maintain normal photosynthetic efficiency and chlorophyll content in zoysiagrass.

4-Coumarate-CoA ligase (4CL) is an important branch point involved in phenylpropanoid metabolism that regulates the biosynthesis of flavonoids, lignin and other phenolic secondary metabolites and plays important roles in plant physiology and biotic and abiotic stresses (Lavhale et al., 2018; Chen et al., 2020; Wang et al., 2022). *4CL* genes emerge in response to salt stress in a variety of plants. In desert poplars, the expression of *Pp4CL2, Pp4CL11*, and *Pp4CL12* is increased significantly in response to salt stress compared with salt-sensitive poplar (Zhang et al., 2015). In *Arabidopsis*, higher expression levels of lignin biosynthesis-related genes, including *4CL1* and *4CL2*, are detected in salt-adapted cells with increased lignin content and thickened cell walls relative to normal cells (Chun et al., 2019). In *Sophora alopecuroides*, transcriptomic analysis revealed that *Sa4CL*, which is involved in lignin synthesis, is significantly upregulated under salt stress (Zhu et al., 2021). In our study, a *4CL1* gene was screened between wild-type and *OX-ZmPDI* transgenic plants (Table 1). However, the *4CL1* gene was downregulated after salt treatment in transcriptome sequencing, and its downregulation was more obvious in *OX-ZmPDI* transgenic plants (Table 1), indicating that *ZmPDI* may influence the expression of *4CL1* to regulate the salt tolerance of zoysiagrass. The protein alignment of *4CL1* genes in *Z. matrella* and *Arabidpsis* showed that the amino acid sequences of Zm4CL1 and At4CL1 have an obviously difference (Supplementary Figure 6B). Therefore, the function of 4CL1 in zoysiagrass may be different from that in other plants.

## Data availability statement

The datasets presented in this study can be found in online repositories. The names of the repository/repositories and accession number(s) can be found in the article/Supplementary material.

## Author contributions

QM and KW performed the evaluation experiments. JW, QM, and KW performed the transcriptomic and proteome analyses and verification experiments. JL and JW designed the experiment. XL, PW, HG, JC, and JZ supervised the project. JW and QM participated in writing the manuscript. All authors contributed to the article and approved the submitted version.

## Footnotes

1
http://zoysia.kazusa.or.jp/

2
http://www.rproject.org/
