# Supplementary material for: The combination of RNA-seq transcriptomics and data-independent acquisition proteomics reveals the mechanisms underlying enhanced salt tolerance by the ZmPDI gene in Zoysia matrella [L.] Merr
Source: Front Plant Sci. 2022 Aug 8;13:970651. doi: 10.3389/fpls.2022.970651 (PMC9393727; doi:10.3389/fpls.2022.970651)
Supplement: Supplementary file 1 [file Data_Sheet_1.docx]

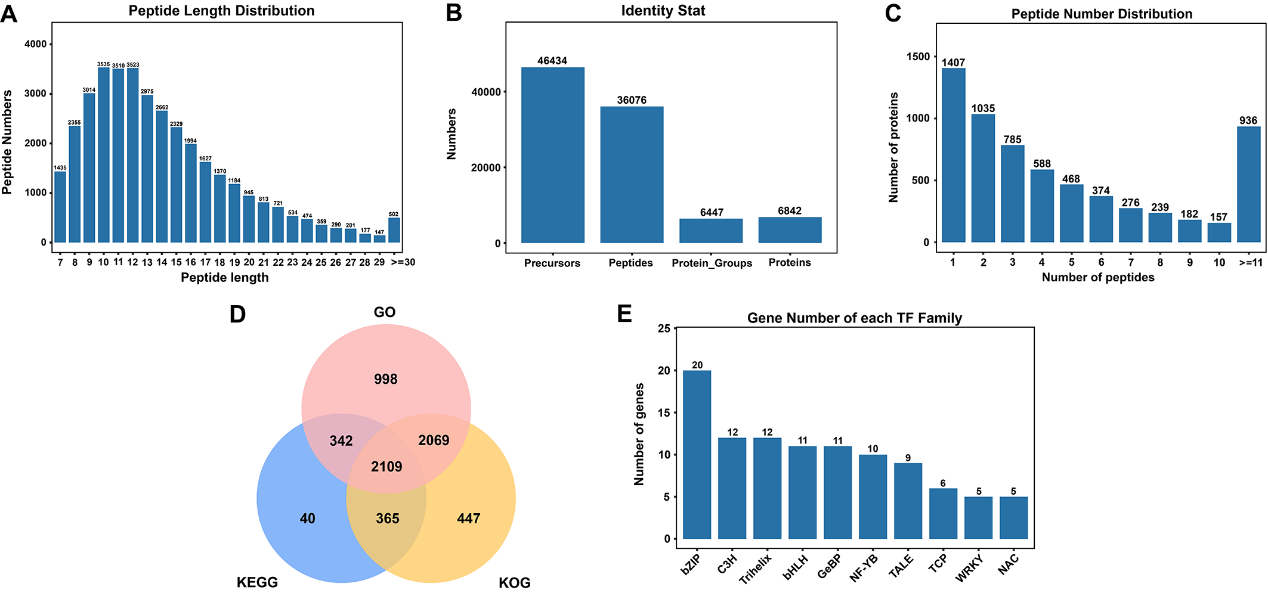


**Figure S1. Protein identification and annotation of the proteome. (A)** Graph of the peptide length distribution. **(B)** Statistical analysis for protein identification. **(C)** Basic statistics for peptide fragments used in protein identification. **(D)** Venn diagram of the number of protein annotations in the GO, KEGG and KOG databases. **(E)** Gene numbers of the top 10 TF families.


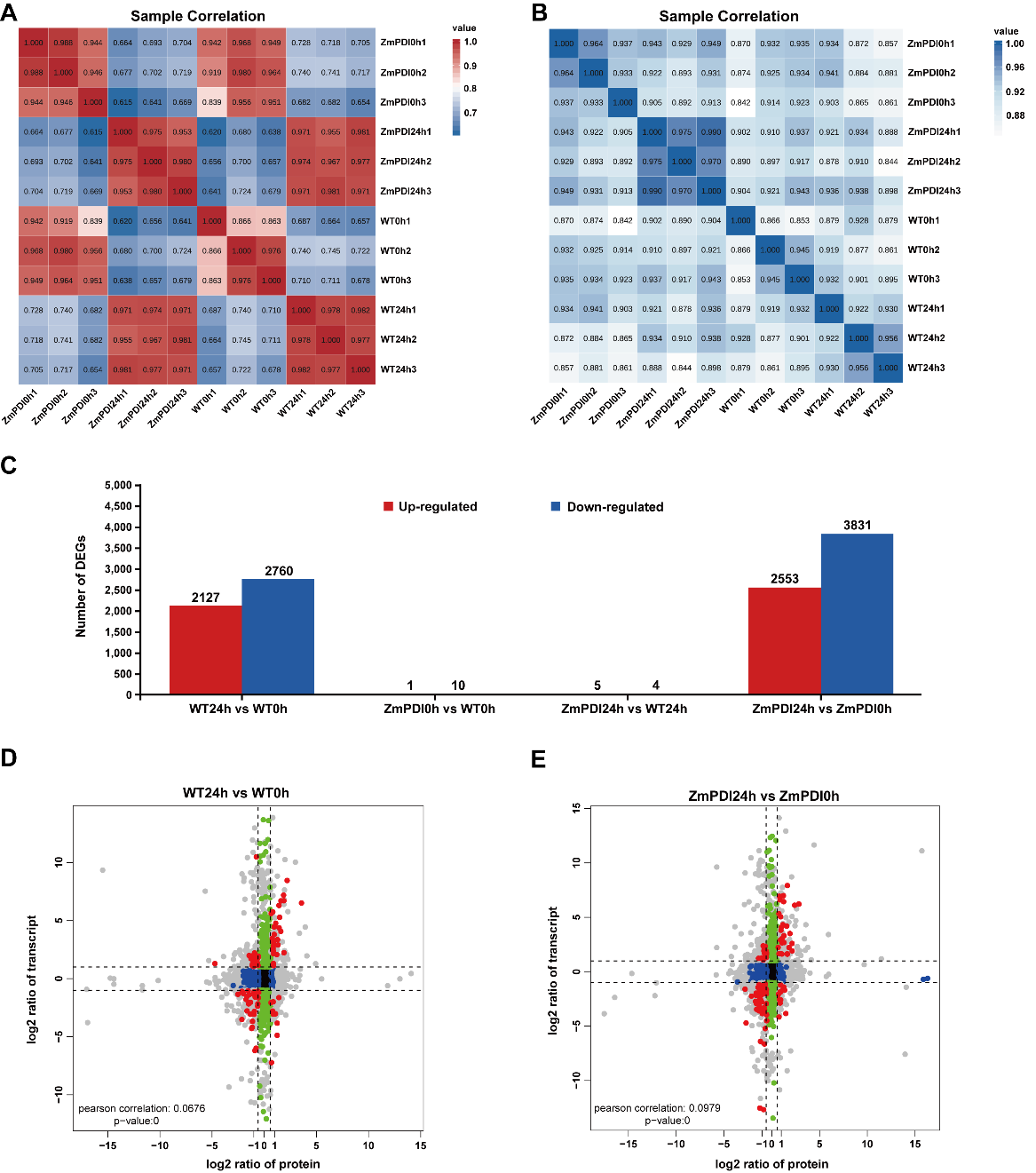


**Figure. S2 Pearson’s correlation and nine-quadrant map analysis of salt tolerance-related transcriptome and proteome of *OX-ZmPDI* transgenic and WT plants of *Z. matrella*. (A)** Pearson’s correlation between 12 samples based on RNA-seq analysis. **(B)** Pearson’s correlation between 12 samples in proteome sequencing. **(C)** The number of up- and downregulated DEGs in WT24h vs. WT0h, ZmPDI0h vs. WT0h, ZmPDI24h vs. WT24h and ZmPDI24h vs. ZmPDI0h. **(D)** Nine-quadrant map of transcriptome and proteome combination analysis in WT plants after salt treatment. **(E)** Nine-quadrant map of transcriptome and proteome combination analysis in *OX-ZmPDI* transgenic plants after salt treatment.


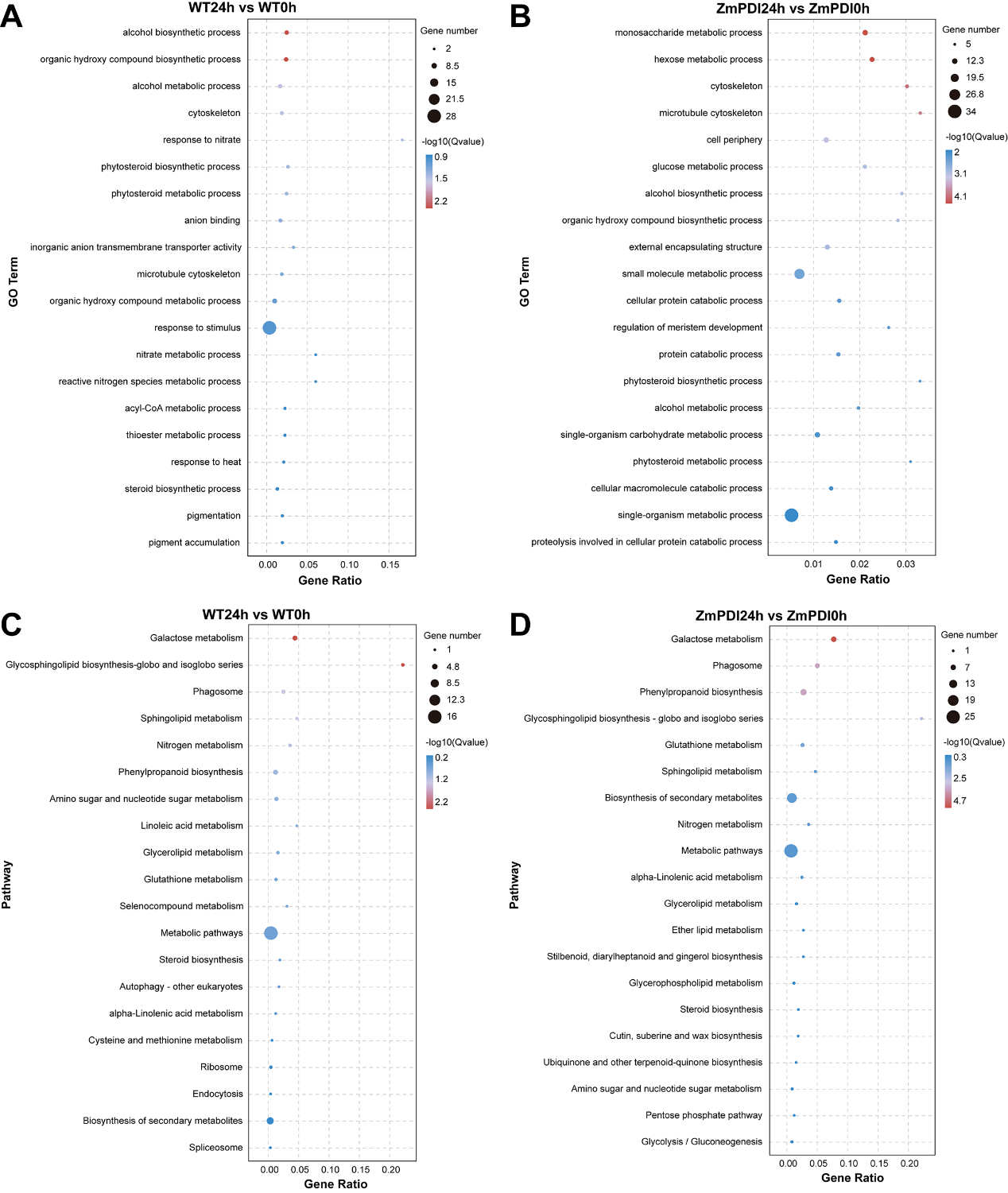


**Figure S3. Top 20 enriched GO terms and KEGG pathways of DEGs that met the P value threshold in quadrants 3 and 7. (A)** The top 20 enriched GO terms of DEGs met the P value threshold in quadrants 3 and 7 in the WT24h vs. WT0h comparison. **(B)** The top 20 enriched GO terms of DEGs met the P value threshold in quadrants 3 and 7 in the ZmPDI24h vs. ZmPDI0h comparison. **(C)** The top 20 enriched KEGG pathways of DEGs met the P value threshold in quadrants 3 and 7 in the WT24h vs. WT0h comparison. **(D)** Top 20 enriched KEGG pathways of DEGs that met the P value threshold in quadrants 3 and 7 in the ZmPDI24h vs. ZmPDI0h comparison.


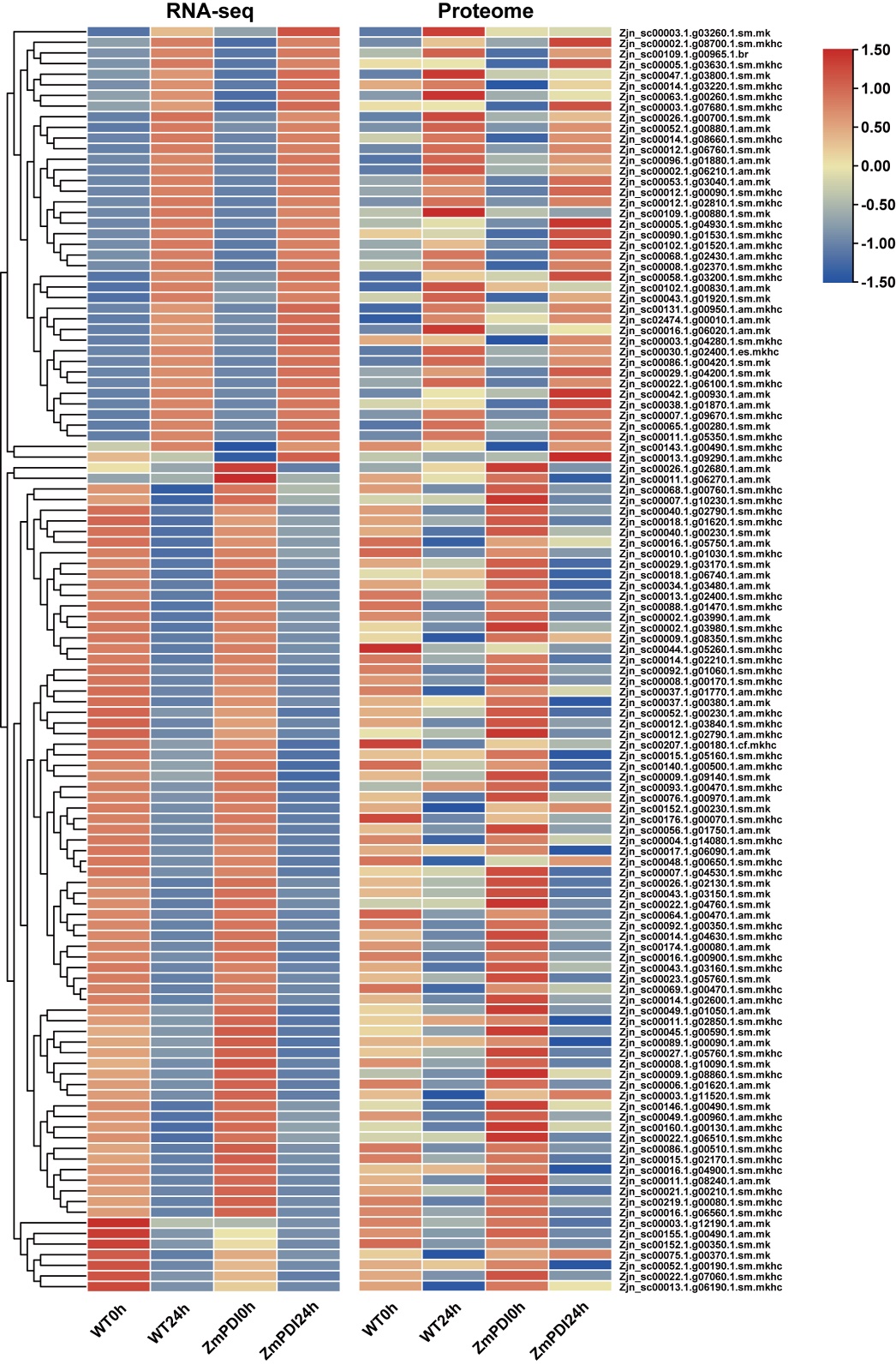


**Figure S4. Heatmap of 119 DEGs in quadrants 3 and 7 of the WT24h vs. WT0h and ZmPDI24h vs. ZmPDI0h comparisons.**


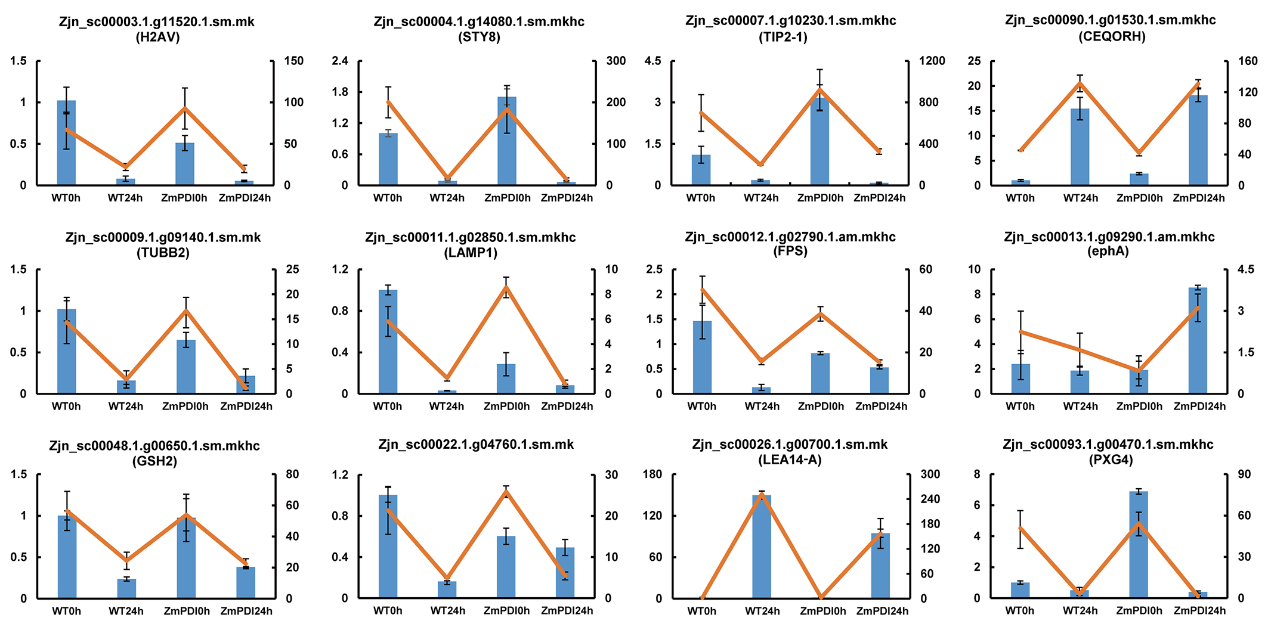


**Figure S5. qRT–PCR validation of 12 genes randomly selected from the 36 DEGs in Table 1.** Values are presented as the mean ± SE. The column diagrams represent the relative expression levels of genes, and the line charts represent the FPKM values of genes.


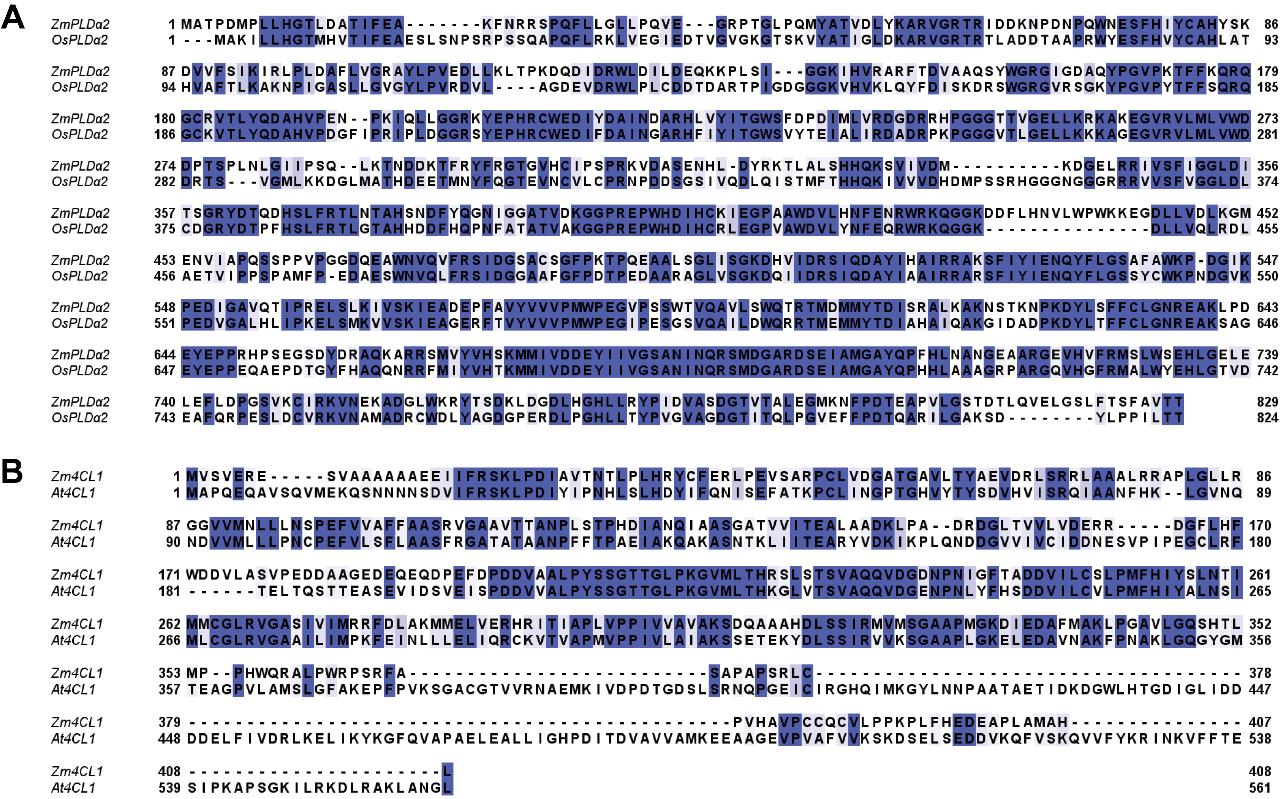


**Figure S6. Protein alignment of *PLDα2* and *4CL1* genes. (A)** Protein alignment of *PLDα2* genes in *Z. matrella* and *O. sativa*. **(B)** Protein alignment of *4CL1* genes in *Z. matrella* and *A. thaliana*. Dark blue represents 100% identity.
